# Ten simple rules for teaching data science

**DOI:** 10.1371/journal.pcbi.1014338

**Published:** 2026-06-10

**Authors:** Tiffany A. Timbers, Mine Çetinkaya-Rundel

**Affiliations:** 1 Department of Statistics, The University of British Columbia, Vancouver, British Columbia, Canada; 2 Department of Statistical Sciences, Duke University, Durham, North Carolina, United States of America; Dassault Systemes BIOVIA Ltd, UNITED STATES OF AMERICA

## Introduction

Data science is the study, development, and practice of using reproducible and transparent processes to generate insight from data [1–4]. With roots in statistics and computer science, data science educators draw on many of the teaching strategies used in those fields [5–7]. However, data science is a distinct discipline with its own unique challenges and opportunities for teaching and learning. Here, we collate and present ten simple rules for teaching data science, piloted by leading data science educators in the community and successfully applied in our own data science classroom. We believe these rules are relevant for data science instructors teaching in both formal credit courses and workshop settings.

## Rule 1: Build a safe, inclusive, and welcoming community

The first rule is to build a safe, inclusive, and welcoming community. The reason is that people don’t learn effectively when they don’t feel psychologically safe. Psychological safety is the belief that one can express oneself, through speech or actions, without fear of negative consequences or feedback [8]. If learners do not feel safe asking questions without being made to look or feel dumb, they are not going to ask questions or be engaged [9]. If they don’t feel safe from negative perceptions about their intelligence, discrimination, or harassment in the classroom (or related spaces such as office hours, course forums, study groups, etc.), they may become so disengaged that they even stop showing up. Thus, creating safe, inclusive, and welcoming learning environments is crucial for effective learning, and as instructors, we have a responsibility to establish scaffolding and guidelines that facilitate this.

One thing we do in our courses is establish a course code of conduct, which holds prominence in the classroom and related learning spaces. At the beginning of a new course, we take time in the first class to present our code of conduct to the learners. The codes of conduct we use are very explicit. They discuss expected behaviors, behaviors that will not be tolerated, the process for reporting violations, and the consequences for violating these rules. We also ensure that there are multiple ways to report violations, to support students in the unlikely event that the instructor is the one violating the code of conduct. It is also important that, when a violation is reported, it be taken seriously and addressed in a timely manner. No student concern should ever be ignored or brushed off. For instructors seeking to establish a code of conduct for their course, we recommend examining existing codes of conduct from other organizations and tailoring them to your specific context. One particularly good example is The Carpentries’ code of conduct (https://docs.carpentries.org/topic_folders/policies/code-of-conduct.html).

This rule is likely important for teaching any subject, but we strongly feel that it must be included here as a first, and perhaps a ground rule, for data science in particular, for two reasons. First, data science involves extensive interaction and collaboration in online spaces. It is well documented that people are less inhibited in their online interactions, and that this disinhibition can manifest in toxic forms, including rude language, harsh criticisms, expressions of anger or hatred, and even threats [10]. Second, at present, data science suffers from significant deficits in diversity, equity, and inclusion [11]. Each of these factors independently poses challenges to creating safe, inclusive, and welcoming learning environments; in combination, their effects are compounded. That is why we believe it is essential to be proactive in establishing behavioral norms from the outset of a course. This is not merely good pedagogical practice; it is an act of structural intervention in a field that has historically failed to welcome many of the people who deserve to be in it.

## Rule 2: Teach data science by doing data analysis

The second rule is teaching data science by doing data analysis. This means that in your first data science lesson, not your third or 10th lesson, have students load data, perform simple data wrangling, and create a data visualization. We call this the “let them eat cake” approach to teaching data science [12].

Why do we suggest this? Because, just like eating cake, it’s extremely motivating to students. Most students sign up for a data science course or workshop because they’re interested in asking and answering questions about the world using data, or because it’s a degree requirement. In either case, they likely don’t yet have sufficient knowledge to care deeply about detailed, technical aspects, such as object data types, whether to use R versus Python, or, if using R, whether to opt for the tidyverse or base R. As a result, we should show them something interesting very early on to hook them – this is especially important for the group who are taking the course primarily because it is a degree requirement.

After they are hooked, they will be begging you to answer questions about the detailed, technical aspects you intentionally omitted. An example of this is the first chapter of *Data Science: A First Introduction* [4], a textbook one of the authors uses to teach introductory data science. There, we ask learners to load data from a CSV file and perform introductory data wrangling using filtering, arranging, and slicing. Finally, create a plot to answer a question about indigenous languages in Canada: how many people living in Canada speak an indigenous language as their mother tongue? Other leading data science educators who advocate and practice this rule include Wang and colleagues [13], David Robinson in his introductory online Data Science course [14,15], and Jenny Bryan, a software engineer at Posit (formerly RStudio) and renowned data science educator, who summed this up nicely in a tweet “[...] I REPEAT, do not front-load the ‘boring’, foundational stuff. Do realistic and non-boring tasks and work your way down to it.” [16]

## Rule 3: Use participatory live coding

The third rule is to use participatory live coding. This means that when you are working with code in the classroom, instead of showing it on a static slide or just running it in an executable slide or an integrated development environment (IDE; a tool for developing software code), actually type the code and narrate it as you teach. Have the participants follow along as well. The reason is that it gives you the opportunity to demonstrate your best practices for processes and workflows, topics that are important in practice but, unfortunately, are often just an afterthought in teaching computational subjects. You can discuss why you’re approaching things differently as you go. You are also likely to make mistakes as you live-code, and that’s actually a good thing. It helps you appear human to students because they, too, will make mistakes. More importantly, it allows you to demonstrate how you approach debugging to solve code problems, which they can leverage in their homework and later in their work outside the course. Participatory live coding also slows you down, so you don’t go too fast for the students. This pedagogy originates from the “I do, we do, you do” method of knowledge transfer [17], and its application in teaching programming was pioneered by The Carpentries, a global nonprofit (https://carpentries.org). Best practices for doing this have been refined and shared as ten quick tips by Nederbragt and colleagues [18].

## Rule 4: Give tons of practice and timely feedback

The fourth rule is to give tons of practice. Give the learners many, many, many problems to solve, probably many, many, many more than you think they might need. This is because repetition leads to learning [19]. That’s not just in humans; it is more fundamental than that. In animal behavior, repetition is known to lead to learning across the animal kingdom [20,21]. Similarly, students need to practice tasks repeatedly to understand them and perform them effectively. For example, when teaching students to read data from a file, don’t just give them one file to read; instead, have them work with six different variants of a very similar file. With this approach, students have to investigate each file in detail, including checking the file type, column spacing, whether there’s metadata to skip, whether there are column names, etc. In our courses, they will complete these six variants in an in-class worksheet, a lab assignment, and a quiz. Meaning that by the end of the course, they will have practiced this skill over 15 times. Many excellent data science educational resources use this pedagogy, including software packages (e.g., the swirl R package [22]), online courses (e.g., Kaggle Learn [23]), and popular textbooks (e.g., R for Data Science [24]). For those new to designing practice exercises for data science, we recommend looking at the “Exercise Types” chapter of *Teaching Tech Together: How to make your lessons work and build a teaching community around them* by Greg Wilson [25].

An excellent question instructors may ask in response to reading this rule is, “How do we make time in class and across the curriculum for practice as well as knowledge dissemination?” One strategy we often employ is assigning readings or videos as pre-class homework rather than using the entire class time for lecturing. Allowing time for in-class practice is extremely valuable, as instructors and teaching assistants are present to help students get unstuck when they run into trouble. This is often referred to as flipping the classroom [26]. We also recommend taking a critical look at the list of topics to be taught and asking the hard question of what is essential for that course or workshop. As there is strong evidence that little to no learning occurs without practice and repetition, there seems to be little point in teaching topics when no time is allotted for practice.

When giving lots of practice, pair it with timely feedback. Practice without feedback has limited value. So, how can we provide a wealth of opportune feedback, especially with our limited teaching capacity and resources? One way we can do this is through automated software tests. In data science, many of the problems we assign to students involve writing code. As a consequence, we can write software tests that provide feedback to students, letting them know when they give an incorrect answer in a particular way, as well as offering a gentle, helpful nudge to solve the problem in a different way.

**Fig 1:**
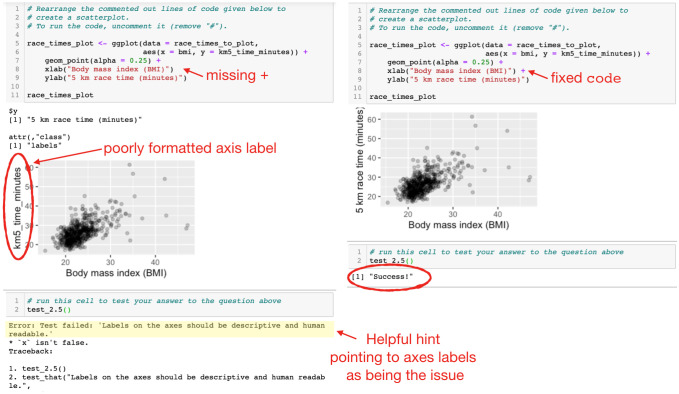
Example of automated software test feedback to students.

Fig 1 shows an example of this in practice. Here, students were given some ggplot code in a Parsons problem format (the lines of code were given in the wrong order, and the students needed to rearrange them to the correct order). In this example, a student has rearranged the code but, while doing so, has introduced a syntax error. As a result, a plot is created, but it’s not quite what we expect. Without timely feedback, the student might not realize that there’s a problem with their code until much later, or not at all if they fail to check the feedback and solutions after the assignment is graded and grades are returned (often days or weeks later).

Automated software testing can provide timely feedback while students’ focus and attention are on the topic being learned and practiced. This pedagogy was first developed and used for teaching programming in computer science courses [reviewed in 25] and is now being adopted in data science courses. There are now many wonderful and popular software packages for this in the context of data science, including the learnr R package [27] for R code and the NBgrader [28] and Otter Grader [29] packages, which work with both R and Python code.

Most recently, advances in generative artificial intelligence (AI) have provided another tool for providing students with timely feedback: large language models (LLMs). There is much excitement and promise in using LLMs in this way for teaching, as they are more flexible than software tests and can provide feedback on narratives and visualizations, in addition to code. One of the authors of this paper has recently piloted this, and many other educators across fields are also exploring it (as evidenced by a growing body of literature on LLMs in education). One note of caution for instructors interested in heading out on this brave new journey is the high risk of inequitable access to models. Using LLMs often requires a paid subscription. In a university setting, unless the institution has a site license or locally hosts open source models and provides unlimited access to them for its students, this can create equity issues for students who cannot afford these tools. As the tooling around LLMs continues to mature rapidly, instructors who invest in learning about LLMs and how to use them are likely to be well-positioned to make informed decisions about when and how to adopt these tools as they evolve. This connects naturally to our final rule: *Keep up to date with modern tooling*.

## Rule 5: Use tractable or toy data examples

Our fifth rule is to use tractable or toy data examples when introducing a new tool, method, or algorithm to students. Tractable or toy data sets have a countable number of elements, which can fit in our working memory. This allows students to track the progression of everything through the algorithm’s steps, see how the elements are manipulated, and gain a deeper understanding of these concepts. For example, in one of our courses, we use the Palmer Penguins dataset [30] to introduce k-means clustering. Instead of giving them the entire data set, which contains hundreds of observations, we first subset it to just a handful. Then we can walk the students through what happens to these observations at each step of the algorithm, building their understanding and intuition. Inspiration for this comes from the STAT 545 dplyr joins cheat sheet [31], which covers various joins in the dplyr package [32]. This is a difficult topic for students to wrap their heads around, and when instructors teach it using large datasets, it is really hard for students to see the differences between the types of joins and to understand how they work. To make matters worse, all of these joins are also similarly named (e.g., left join, right join, inner join, outer join, etc). The cheat sheet addresses this issue by using two toy data sets on superhero comic characters and publishers; the superhero dataset has only 7 rows and 4 columns, while the publisher dataset has 3 rows and 2 columns. The cheat sheet outlines all the possible joins, narrating and displaying the output of each. These data sets are small enough to fit in learners’ working memory, making the joins tractable and much easier to understand.

It is, of course, not feasible to keep learners interested and motivated to learn more if we rely solely on toy datasets. So, once students have a conceptual understanding of the tool, method, or algorithm being taught, we can move on to using real, rich datasets, which is our next rule.

## Rule 6: Use real and rich, but accessible data sets

After you’ve helped students develop a conceptual understanding of the new tool, method, or algorithm you are teaching, the next step is to have students apply it to realistic questions and real, rich data. However, as you do this, it is critical to ensure the dataset is also accessible to all your learners. The question, as well as the observations (i.e., rows) and variables (i.e., columns) in the dataset, must be things that your learners can quickly understand.

This can easily become an expert blind spot for us when we are teaching, especially if we have training in a particular domain. Educators, like all experts, have blind spots, whereby they tend to teach from the subject’s deep principles rather than building on the learner’s more basic knowledge [33]. For example, one of the authors, who is trained in the biological sciences, might think that using a deep sequencing data set would be a great and motivating example for a particular algorithm we want to teach students. However, this thought likely stems from the author’s deeper understanding of biological processes, which, in this example, gives them an expert blind spot. Given that, if learners do not have similar background knowledge, that dataset might not be appropriate. It might introduce too much cognitive load for the students, to the extent that they cannot focus on the task at hand — refining their understanding and application of the tool, method, or algorithm being taught.

We do not want to use up our students’ limited cognitive resources to understand the dataset. Instead, we want to use something that all our learners can easily understand, including what the observations are and what the columns represent. One example from one of the courses we teach uses Canadian census data on languages spoken across different regions [34]. Other examples include the Gapminder [35] and UN Votes [36] data sets. These are particularly nice examples because they contain hundreds of observations; however, the observations are something most people can understand: a country, a year, a population, a vote, etc. And with such a dataset, we can ask questions that most learners are interested in, because everyone has grown up in at least one country, and possibly more, in their lifetime. And we all grew up at different times in history. These lived experiences make us generally knowledgeable and inclined to ask questions about the datasets.

There are many more real, rich datasets that are accessible and usable. The main point here is that it shouldn’t take your students long to understand the data set, because a deep understanding of it isn’t what you are trying to teach at that moment. Beyond accessibility, it is also worth considering relevance: varying topics across a course ensures that all learners encounter data they find genuinely interesting, and incorporating data that reflects your learners’ local context (e.g., country, region, culture) and/or current events can deepen that connection further. It can be difficult to find such data sets in a timely manner, but it’s often worth the effort for learners to connect what they are learning in their data science course to what’s happening in their own lives and communities outside the classroom.

## Rule 7: Provide cultural and historical context

Our seventh rule is to provide a cultural and historical context for what you’re teaching. To illustrate this rule, we will provide examples from the historical context of the field of statistics as well as the cultural context of software features.

For example, if you decide to teach a data science topic using Anderson’s well-known iris data set, it is critically important to educate students about the first published use of this data set by R. A. Fisher in the Annals of Eugenics [37]. There, he used the iris data set to advance eugenic methodology. Thus, the iris data set has a documented history in eugenics research, a difficult fact to address rather than sweep under the rug. Alternatively, even if educators choose to use a more modern alternative dataset (e.g., the Palmer Penguins dataset [30]), it is still important to discuss the field’s difficult history, which matters regardless of which dataset you use. Eugenics caused real harm to real people, and statistics played a role in that history. These conversations are not always easy, and few of us received explicit training in how to hold them. But acknowledging the harm caused by eugenics and its place in the history of our field is part of responsible teaching. Helping students understand this history is how we can equip them to think critically about the methods and data they inherit. It also helps ensure new generations do not repeat the mistakes of the past.

This rule also applies when teaching a new software tool or a new feature of a tool they are familiar with, whose design does not seem optimal from the learner’s perspective. Rather than leaving students to feel confused or frustrated, it is helpful to explain why the tool was built the way it was. If you give the design and historical context, for example, saying people thought about this when they built the tool and decided this was the best way to implement it for reasons X, Y, and Z, it helps the students understand that software tools are built by humans, and so they are going to be influenced by humans’ perspective, human history, and human culture. Furthermore, it helps prevent frustration or annoyance with the software by allowing users to rationalize why it works the way it does. We believe it is crucial to help prevent these frustrations and annoyances, as we have observed that for some learners, they can become significant barriers and lead to dislike or avoidance of a particular piece of software.

For example, when we teach the programming language R [38], we use the suite of tidyverse R packages [39]. Learners observe that these packages heavily use unquoted column names when referencing data frame columns in their function calls. That is really strange when you come from other programming languages, as most other programming languages require quoted strings when referencing object attributes (which is what a data frame column is). This can also make writing functions that utilize tidyverse functions a bit more challenging due to issues with indirection. For learners with experience in Python, Java, or C, this might initially seem like a really bad idea. However, once they learn that R and the suite of tidyverse R packages were written by statisticians for performing data analysis and graphics [38,39], and that they designed the language and packages with the expectation that much of the users time would be frequently typing things into the console or running code interactively, it makes a lot more sense that they would want to minimize the amount of typing and tracking of opening and closing quotations for their users. Doing this minimizes the potential syntax errors that could be introduced by forgetting to open or close quotation marks.

Another example is the use of the arrow (<-) as an assignment operator in R, which may seem odd to some learners because it uses two characters. Again, however, when given the historical context that R was derived from another programming language named S, and S was inspired by another programming language, APL, that was designed for a particular keyboard with one key mapped to the assignment operator [40], it makes a lot more sense as to why that design choice was made. Design and historical contexts help learners understand the rationale behind different design choices, allowing them to see choices they might not initially agree with as excellent within these contexts. As an aside, for those interested in learning more about the history of the R programming language, see the “History and Overview of R” chapter in [41].

## Rule 8: Use checklists to focus and facilitate peer learning

Rule number eight is to use checklists to focus and facilitate peer learning. A well-documented pedagogical best practice is for peers to learn from each other [42]. One implementation of this is peer review. In peer review, learners benefit from receiving feedback on their own work before submission. This gives them the opportunity to learn and work to improve it. Learners also benefit from reviewing others’ work, as they will see examples of what they are working on, some stronger and some weaker than their own. This helps them refine their vision of what “good” looks like for the task they are working on.

However, peer review can be particularly challenging if you haven’t done it before, or if it involves reviewing something new that you are still learning. So what can we, as instructors, do to facilitate this practice for our learners? Given that we are well-practiced at creating rubrics for grading student work, we can use our existing rubrics as a starting point for drafting peer-review checklists.

Why checklists? Checklists can help ensure complex tasks are completed successfully and have been used in safety-critical systems (such as aviation, surgery, or nuclear power). They can be particularly helpful for completing complex tasks, as well as for repetitive ones that can become boring [43]. For these reasons, checklists have recently been adopted in scientific (e.g., PLoS journals, Nature Ecology & Evolution, Journal of Open Source Education, Journal of Open Source Software) and software (e.g., ROpenSci, PyOpenSci) publishing to help ensure that reviewers and editors increase transparency, decrease bias, and call attention to essential elements of reviews that are often overlooked [44]. As reviewers for some of these journals and organizations, the authors have found checklists extremely helpful for focusing our attention on the most important elements of a review and for ensuring we don’t miss anything. We believe this idea also has value in training data science students.

**S1 Checklist** shows an example of a data analysis review checklist that we have used in our data science courses. It serves to communicate the aspects that we, as educators, believe are important for the assessment to be completed to a high quality. In addition to checking off the checklist items, students are also asked to provide written comments and feedback. This checklist helps focus students’ comments and feedback on the items they were unable to check off. For example, if there were issues with the software tests (e.g., they were missing) and issues with the discussion section (e.g., they did not mention any limitations of the analysis), they would not check those boxes, and that would help focus their review comments on critical feedback about the issues with those sections.

## Rule 9: Teach students to work collaboratively

Data science is a highly collaborative discipline. Data scientists often work in teams or, at a minimum, need to collaborate with other stakeholders (e.g., domain experts, project managers, clients). Consequently, it is essential that we teach our students to work collaboratively. To do this effectively, we must teach them both the technical tools and skills necessary for collaboration (e.g., version control tools such as Git and GitHub, project boards) and the social practices that make collaboration effective (e.g., active listening, giving critical feedback, code review). We understand that adding collaboration to an already full curriculum requires difficult decisions about what to drop. We argue, however, that foundational collaborative skills are as important for students as traditional statistical topics, though the emphasis will ultimately depend on the goals of the course.

We must also give them opportunities to practice collaboration. Practice should occur at increasing levels of complexity, starting with smaller in-class activities, such as think-pair-share [45] and pair programming [46], progressing to larger group assignments, and culminating in projects. By “projects,” we mean an entire data product (ideally on a topic of their choosing, if feasible) from beginning to end.

Projects give students the opportunity to experience the entire data science workflow. Doing project work is important because it effectively motivates students. It also provides students with valuable experience in dealing with the messiness of real data (we all know that in the real world, data is often quite messy). Additionally, courses typically focus on just a small part of a data analysis workflow at a time. For example, they might focus on data visualization, data wrangling, or modeling. But in a project, they get to see how all these pieces fit together in a more realistic scenario. This is critical training for any aspiring data scientist [47].

From the instructor’s perspective, however, projects can feel daunting, particularly when student numbers are large and teaching resources are not. One way to make projects more feasible is to scope them. You may want to limit the project to a particular topic, or have students choose a data set from a given list, use a specific method, or employ a particular programming language. Doing this provides some consistency in grading and allows you to create a rubric that applies to all projects. An example of a scoped project from a course on collaborative software development in data science that we teach asks students to create a Python package with n functions, where n is the number of students in the project group. The functions must be related to a common theme and fall under the umbrella of data science. Students must utilize the packaging tools and collaborative practices taught in our course. This kind of project is very feasible to grade because its scope is well-defined and limited. However, it also allows students to be creative and work on something they are interested in. Another way to scope projects is to have students work on projects that use data related to their own research or thesis work, but utilize the data science concepts, tools or techniques being taught in the course.

When getting students to work collaboratively on longer-scale assignments and projects, it is important to scaffold good collaboration practices into your assignment or project expectations (and grading). In our project courses, we have students spend the first working session almost entirely on group formation activities. In this session, we have them participate in icebreaker activities to get to know each other and build trust. After that, we have them create a team contract outlining their expectations for the project and how they will work together. During the project, we have them hold regular team check-ins (e.g., meetings, written updates, and progress reports) and use project boards to track their tasks and progress. At the end of the project, we have them complete a teamwork reflection activity to discuss what went well and what could be improved. As seasoned collaborators, we often take these practices for granted. However, for students new to collaboration, these practices are often missed, and as a result, collaboration frequently breaks down. Even with these scaffolds in place, we still see some groups struggle with collaboration. To manage this, we recommend discussing with students the importance of expecting, rather than avoiding, conflict, and to encourage them to work as a group to develop a plan ahead of time for how to manage conflict it when it arises. Beyond Intractability, an online knowledge base on resolving intractable conflicts, offers a useful guide to conflict resolution for students working on group projects [48].

## Rule 10: Keep up to date with modern tooling

And finally, our tenth rule is to keep up to date with modern tooling. Data science is a rapidly evolving field, and new tools and technologies are constantly emerging. Educators should stay informed about developments in data science tools and incorporate relevant ones into teaching. This helps ensure students gain in-demand skills and allows instructors to enhance their teaching with new features. How can data science educators keep up with modern tooling?

The first step is to stay informed. Data science educators need to be actively connected to and engaged with data science communities so they can stay informed about new developments in the field in a timely manner. There are many ways to do this, including attending in-person or virtual events (e.g., meetups, conferences), reading blogs, preprints, and papers, and following data scientists on social media. Educators do not need to do all we list, and they should choose the method(s) that best fit them. What is most important is that data science educators must not become siloed.

The second step is for data science educators to incorporate the new methods and tools into their own workflows to learn about them more deeply and experience them. This could mean using the tool in their research, or even if the instructor is not a researcher, they can and should test the tool out in their data science teaching workflows. This allows educators to develop more informed decisions and opinions about the new tool, which they can share with their students when teaching about it. Doing this also helps build the instructor’s credibility and ability to answer questions about them. Once well-informed and experienced with the new tool, data science educators must finally evaluate and carefully consider how (and if) to use and teach new modern tooling by considering its place in the data science ecosystem and in society, culture and human (and environmental) ethics. These considerations should be shared with students when teaching new tooling, as well as when asking them not to use particular new tooling or alternatives to what may be the most popular mainstream option at the time.

At the time of writing, a timely example is generative artificial intelligence (AI), including both chatbots and agentic code-generation tools that can write, debug, and suggest code. Students are already using these tools, often not in the most effective ways, such as taking shortcuts that undermine their learning. Yet these tools also offer real opportunities: more timely feedback, support for debugging, and practice with tools increasingly standard in industry and academia. The landscape is complicated, however. First, unlike the other tools discussed in this paper, generative AI tools — particularly agentic ones — often require paid subscriptions, raising equity concerns in university and workshop settings, where not all learners can afford access. Second, ethical and privacy concerns mean some students may reasonably choose not to use them, a choice that should carry no penalty. Third, the line between AI-assisted work and plagiarism remains blurry, creating grading challenges for instructors. Without staying informed and experimenting with these new generative AI tools, data science educators cannot make sound pedagogical choices about how to incorporate them into the classroom and their teaching, nor can they engage students in open, honest discussions about their ethical and practical implications.

## Conclusion

This list of ten simple rules for teaching data science is by no means exhaustive, but we hope it provides a useful starting point for new data science educators. This list was curated from our own experiences teaching data science, as well as from what we’ve seen other leading data science educators practice.

## Supporting information

S1 Checklist Data analysis peer-review checklist. A checklist for peer review of data analyses used in data science courses taught by the authors.
(PDF)
