# Supplementary material for: Ten simple rules for teaching data science
Source: PLoS Comput Biol. 2026 Jun 10;22(6):e1014338. doi: 10.1371/journal.pcbi.1014338 (PMC13252730; doi:10.1371/journal.pcbi.1014338)
Supplement: S1 Checklist — A checklist for peer review of data analyses used in data science courses taught by the authors. (PDF) [file pcbi.1014338.s001.pdf]

Data analysis review checklist

Reviewer:

Conflict of interest

- ☐ As the reviewer, I confirm that I have no conflicts of interest for me to review this work.

Code of Conduct

- ☐ I confirm that I read and will adhere to the MDS code of conduct.

General checks

- ☐ **Repository:** Is the source code for this data analysis available? Is the repository well organized and easy to navigate?
- ☐ **License:** Does the repository contain a plain-text LICENSE file with the contents of an OSI approved software license?

Documentation

- ☐ **Installation instructions:** Is there a clearly stated list of dependencies?
- ☐ **Example usage:** Do the authors include examples of how to use the software to reproduce the data analysis?
- ☐ **Functionality documentation:** Is the core functionality of the data analysis software documented to a satisfactory level?
- ☐ **Community guidelines:** Are there clear guidelines for third parties wishing to 1) Contribute to the software 2) Report issues or problems with the software 3) Seek support

Code quality

- ☐ **Readability:** Are scripts, functions, objects, etc., well named? Is it relatively easy to understand the code?
- ☐ **Style guidelides:** Does the code adhere to well known language style guides?
- ☐ **Modularity:** Is the code suitably abstracted into scripts and functions?
- ☐ **Tests:** Are there automated tests or manual steps described so that the function of the software can be verified? Are they of sufficient quality to ensure software robustness?

Reproducibility

- ☐ **Data:** Is the raw data archived somewhere? Is it accessible?
- ☐ **Computational methods:** Is all the source code required for the data analysis available?

- ☐ **Conditions:** Is there a record of the necessary conditions (software dependencies) needed to reproduce the analysis? Does there exist an easy way to obtain the computational environment needed to reproduce the analysis?
- ☐ **Automation:** Can someone other than the authors easily reproduce the entire data analysis?

Analysis report

- ☐ **Authors:** Does the report include a list of authors with their affiliations?
- ☐ **What is the question:** Do the authors clearly state the research question being asked?
- ☐ **Importance:** Do the authors clearly state the importance for this research question?
- ☐ **Background**: Do the authors provide sufficient background information so that readers can understand the report?
- ☐ **Methods:** Do the authors clearly describe and justify the methodology used in the data analysis? Do the authors communicate any assumptions or limitations of their methodologies?
- ☐ **Results:** Do the authors clearly communicate their findings through writing, tables and figures?
- ☐ **Conclusions:** Are the conclusions presented by the authors correct?
- ☐ **References:** Do all archival references that should have a DOI list one (e.g., papers, datasets, software)?
- ☐ **Writing quality:** Is the writing of good quality, concise, engaging?

Estimated hours spent reviewing:

Review Comments:

Please provide more detailed feedback here on what was done particularly well, and what could be improved. It is especially important to elaborate on items that you were not able to check off in the list above.

Attribution

This was derived from the JOSE review checklist.
